# A Tale of Three Spectra: Basic Symptoms in Clinical-High-Risk of Psychosis Vary Across Autism Spectrum Disorder, Schizotypal Personality Disorder, and Borderline Personality Disorder

**DOI:** 10.1093/schizbullopen/sgae017

**Published:** 2024-07-27

**Authors:** James C Martin, Scott R Clark, Simon Hartmann, K Oliver Schubert

**Affiliations:** Discipline of Psychiatry, Adelaide Medical School, The University of Adelaide, North Terrace, Adelaide, South Australia, Australia; Discipline of Psychiatry, Adelaide Medical School, The University of Adelaide, North Terrace, Adelaide, South Australia, Australia; Discipline of Psychiatry, Basil Hetzel Institute, Woodville, South Australia, Australia; Discipline of Psychiatry, Adelaide Medical School, The University of Adelaide, North Terrace, Adelaide, South Australia, Australia; Discipline of Psychiatry, Adelaide Medical School, The University of Adelaide, North Terrace, Adelaide, South Australia, Australia; Division of Mental Health, Northern Adelaide Local Health Network, SA Health, South Australia; Headspace Early Psychosis, Sonder, South Australia

**Keywords:** basic symptoms, schizotypal personality disorder, autism spectrum disorder, borderline personality disorder, clinical-high-risk, basic self

## Abstract

**Background and Hypothesis:**

The clinical-high-risk (CHR) approach was developed to prevent psychosis through the detection of psychosis risk. CHR services are transdiagnostic in nature, therefore the appropriate management of comorbidity is a central part of care. Differential diagnosis is particularly challenging across 3 common comorbidities, schizotypal personality disorder (SPD), autism spectrum disorder (ASD), and borderline personality disorder (BPD). Phenomenological research indicates a disturbance of “basic self” may differentiate between these commonly comorbid disorders and can be captured by Huber’s basic symptoms (BS) concept. We investigated whether BS vary across these disorders and may inform differential diagnosis in young person’s meeting CHR criteria.

**Study Design:**

A total of 685 participants meeting CHR criteria from the NAPLS-3 cohort completed the COGDIS items of the schizophrenia proneness instrument, a measure of BS, as well as the structured interview for DSM-5 (SCID-5). A logistic regression model was used to investigate the variation of COGDIS across SPD, ASD, and BPD, while controlling for age and SIPs positive severity.

**Study Results:**

Meeting COGDIS criteria was positively associated with SPD (OR = 1.72, CI = [1.31–2.28], *P* = .001) but not ASD nor BPD.

**Conclusions:**

Our results indicate that “basic self-disturbance” as indicated by COGDIS differs across SPD, ASD, and BPD. COGDIS may be useful to inform the management of comorbidities in CHR services, by providing insight into subtle subjective experiences that may benefit from disorder-specific interventions.

## Introduction

The clinical-high-risk (CHR)/ultra-high-risk (UHR) approach was developed to screen for psychosis risk in individuals seeking help for mental ill-health. The ultimate goal of the UHR/CHR approach is to prevent the onset of psychosis through the early detection of risk, with the aim of ameliorating a person’s functional decline. While psychosis prediction remains a crucial target for research and clinical practice^1,2^ and substantial progress has been made,^2–4^ less attention has been given to patient outcomes following CHR “treatment”. For example, in a meta-analysis of 10 studies evaluating CHR intervention, only 6 included a secondary measure of global or social functioning and all but one study reported no significant improvement in functioning at 12 months follow-up.^5^

By design, screening for CHR is transdiagnostic and research suggests that overlapping symptoms and clinical heterogeneity contribute to the variability of outcomes.^6,7^ One systematic review concluded that treating comorbidities should be considered the primary focus of intervention.^8^ Consequently, the detection and management of comorbidities which present alongside psychosis risk, is a key therapeutic objective.^2,9^

Over 3 quarters of CHR subjects report comorbid psychiatric disorders, the most prevalent being major depressive disorder (30%), social anxiety disorder (14%), and schizotypal personality disorder (SPD) (12%).^10^ Two further common comorbidities, autism spectrum disorder (ASD) and borderline personality disorder (BPD), have prevalence rates as high as 11.6% and 11.9%, respectively.^11,12^

Amongst common comorbidities, the identification of SPD, ASD, and BPD may be particularly relevant in CHR services. Unlike mood and anxiety disorders, there is strong evidence to suggest the core features of ASD and BPD are less responsive to pharmacological treatments.^13,14^ Moreover, distinct psychosocial interventions are recommended, such as dialectical behavior therapy for BPD and developmental interventions for ASD.^2,15,16^ In contrast, there is some evidence supporting the use of anti-psychotic medication in SPD, but further research is needed to guide the management of SPD symptoms.^17^

DSM-5-TR defines SPD, BPD, and ASD as each belonging to separate spectra of psychopathology.^18–20^ ASD is recognized as a neurodevelopmental disorder, therefore specialized assessments are used to detect symptoms in early development and diagnosis is often established prior to adolescence.^21^ In contrast, DSM-5-TR places restrictions on the diagnosis of SPD and BPD before 18 years of age and prevents the comorbid diagnosis of SPD and ASD. Yet, recent neurodevelopmental models of SPD and BPD argue that core features can occur from childhood onwards and should be investigated.

In clinical practice, these diagnoses are challenging to differentiate.^22–26^ They often co-occur^27,28^ due to overlapping diagnostic criteria^29–31^ and shared neuropsychological profiles.^31^ The presence of attenuated psychotic symptoms (APS) and robust clinical history has been posited as a meaningful way of discriminating SPD from BPD and ASD,^32^ yet these clinical features can be unreliable.^33^

Within CHR services, young person’s reported clinical histories can be imprecise or incomplete, and differential diagnosis is further complicated by rates of APS that are equally elevated across the diagnostic spectrum.^12,34,35^ For example, in a study of 180 UHR patients, Ryan et al.^36^ concluded that those with comorbid UHR and BPD could not be reliably differentiated from UHR patients without BPD. Similarly, Ribolsi et al.^37^ reported elevated autistic traits in UHR and schizophrenia patients and found a significant positive correlation between autistic symptoms and the severity of formal thought disorder. In CHR services, early detection of disorder-specific symptoms may improve case formulations and prevent delays in accessing tailored interventions.

Phenomenological exploration of self-experience may be a promising avenue for clinicians to distinguish between these complex conditions. Disturbances in self-perception are central to all, but with crucial qualitative differences. Bleuler’s notion of schizophrenia involved a loss of the meaningfulness of reality, resulting in unstable back-and-forth between 2 coexisting worlds, 1 private and 1 public.^38^ In contrast, Kanner’s autism involves a stable sense of being anchored in an inflexible private world that is consistently present across all domains of life,^39^ whereas in BPD there is a profound disturbance of self-identity or “narrative self” especially in interpersonal contexts,^19,40^ with the underlying sense of self-experience remaining largely intact.

Recent advances in psychopathology, captured in brief phenomenological screening instruments, may assist clinicians with these diagnostic and treatment challenges. The basic symptoms (BS) concept, first developed by Huber in the 1970’s, focuses on phenomenological disturbances of subjective perceptual and cognitive “self-experience,” distinct from psychotic features and first-rank symptoms.^41,42^ A subset of BS, termed cognitive disturbances (COGDIS), originate from the Cologne early recognition study and were shown to predict schizophrenia in the prodromal stage.^43^ Whether COGDIS has advantages over other methods (eg, SIPS/SOPS/CAARMS) for prediction of broadly defined psychosis prodrome remains controversial.^44^ Instead, COGDIS may have greater specificity for early stages of schizophrenia spectrum disorders (SSDs).^41^

Contemporary phenomenological research suggests that BS and COGDIS capture disturbances of the “basic self” experience, thought to be specific to SSDs including SPD.^45,46^ In this context, “basic self-disturbance” refers to a shift in or loss of one’s fundamental mode of existing in the world. Although the phenomenon was first systematically described within Huber’s BS construct, and later operationalized within the COGDIS and the cognitive-perceptive (COPER) subgroups of the Schizophrenia Proneness Instrument (SPI), it was refined further by Sass and Parnas toward the concept of self-disorder (SD) and operationalized with the 57-item Examination of Anomalous Self-Experience (EASE).^47^ Hence, BS and SD are interrelated constructs that refer to subtle subjective disturbances of self-experience that cluster in schizophrenia spectrum disorders.^48,49^ A meta-analysis of 15 studies supports both constructs as suitable tools for investigating “basic self-disturbance”^50^ and both cross-sectional and longitudinal findings^51–53^ suggest that even where overlapping items are excluded, a moderate to large correlation exists between COGDIS and the severity of SD, as measured by the total number of items endorsed on the EASE.^54^

Being subjective in nature, BS are difficult to detect from an individual’s behavior and may be missed in broad symptom domains of psychosis, such as those used in SIPS/CAARMS.^55^ There is also increasing recognition that subjective experiences are valuable diagnostic markers within modern neuroscience and psychiatry.^56^ BS may have discriminative value in CHR^57,58^ above and beyond existing screening tools, especially for the differentiation of SPD from ASD and BPD, which also present with disturbances in “self-world” relations, such as weak central coherence and unstable self-identity^40,47,59^

Recently, Nilsson et al.^57^ investigated “basic self-disturbance” in those with an ICD-10 diagnosis of either schizotypal disorder or Asperger’s syndrome. In the schizotypal group, “basic self-disturbance” was 4 times greater on average. The authors highlighted the need for further experimental findings to corroborate results. Yet, despite the potential relevance to clinical practice, to the best of our knowledge, no further findings have been published and hypotheses are yet to be investigated in a CHR sample, where they hold significant potential utility.

In this study, we investigated in the NAPLS-3 CHR cohort, whether clinical assessment of BS using COGDIS may aid in differentiating among SPD, ASD, and BPD within a CHR sample. We characterized COGDIS+/COGDIS− first in the whole sample, then proportions in each comorbidity group of SPD, ASD, or BPD. We hypothesized that in CHR participants, meeting COGDIS criteria would increase the likelihood of meeting diagnostic criteria for SPD but not ASD nor BPD.

## Methods

### Sample

Data obtained from NAPLS-3, a consortium of 9 research centers based in the United States, between 2015 and 2018, comprised 806 participants between ages 12 and 30 years, of whom 96 were controls and 710 were help-seeking individuals referred from health providers, educators, social services, or self-referred following community education efforts. Participants were excluded if they met criteria for current or lifetime axis I psychotic disorder, including affective psychoses, had a recorded IQ less than 70, had a history of a central nervous system disorder, or if diagnostic psychosis-risk symptoms (SOPS) were clearly caused by an axis 1 disorder.^60^ All 710 met CHR criteria as determined by criteria of prodromal symptoms (COPS).^61^ At baseline, only the 685 CHR participants who completed the COGDIS items of the SPI were used in our analysis.

COPs uses the SIPS and the scale for assessment of SOPS to define CHR status. The SCID-5 was administered by trained investigators to establish DSM-5 diagnoses at baseline. Consensus diagnosis was reached for each individual only after both intra-site and inter-site expert consultation. Cross-site reliability on CHR ratings using the SOPs ranged from 0.83 to 0.91 (mean = 0.88), minimal site differences were reported, and interclass correlations were in the excellent range.^60^ Further details regarding SIPS reliability and consensus procedures can be found elsewhere.^60,62^

## Measures

### DSM-5 Diagnosis

DSM-5 diagnosis was assessed using the structured clinical interview for DSM-5 (SCID-5) and both SPD and BPD diagnosis were defined using DSM-5 criteria at intake assessment. ASD diagnosis was defined using a combination of DSM-5 checklist during baseline clinical interview, medical records, and caregiver report of historical diagnosis. When scoring the SCID-5, DSM-5 exclusion criteria for age or differential diagnosis were ignored. For example, those with a diagnosis of ASD could also receive a diagnosis of SPD.

### Basic Symptoms

Basic symptoms were assessed using the COGDIS subscale of the SPI—adult version (SPI-A) and the SPI—child and youth version (SPI-CY). Compared to the SPI-A, the SPI-CY incorporated additional information from parents/caregivers. The COGDIS subscale is formed from 9 items rated on a 7-point severity scale according to their maximum frequency during the past 3 months. COGDIS criteria are then determined by the presence of 2 or more items rated with a severity of greater than or equal to 3 (see Table 1). The COGDIS criteria were developed to provide prognostic accuracy for the risk of transition to psychosis^63^ and whereas other basic symptoms such as disturbances in drive and tolerance to stress are considered nonspecific to schizophrenia, cognitive basic symptoms were found to play a central role.^64^ Furthermore, data from over 300 individuals aged 8 and over suggest good inter-rater reliability and construct validity for all SPI subscales.^65,66^

**Table 1.** COGDIS Symptoms Within the Schizophrenia Proneness Instrument

High-Risk Criterion “*Cognitive Disturbances” (COGDIS)*							
At least any 2 of the following 9 basic symptoms with a score of ≥3 (ie, several times in a month or weekly) within the past 3 months							
Unstable Ideas of Reference							
Disturbances of abstract thinking							
Inability to divide attention							
Thought interference							
Thought pressure							
Disturbance of receptive speech							
Disturbance of expressive speech							
Thought blockage							
Captivation of attention by details of the visual field							

### Transition to Psychotic State

Transition to psychosis was defined as meeting the SIPS presence of psychotic symptoms (POPS) criteria. These criteria require that at least 1 of the 5 SIPS positive symptom scales meets intensity (rated 6) of a psychotic level as well as either a frequency of ≥1 hour per day for 4 days per week during the past month or that symptoms seriously impacted functioning to a level that is “severely disorganizing and/or dangerous to self or others.”^67^

### Other Measures

Intelligence quotient (IQ) was measured using the Wechsler abbreviated scale of intelligence (WASI), where mean IQ within a healthy population is 100 (SD = 15).^68^ At-risk-mental-state was assessed using the COPs criteria,^60,61^ which stratifies participants into levels of risk, with genetic risk and deterioration (GRD) representing the lowest risk group, followed by APS, then brief intermittent psychotic symptoms (BIPS). The Calgary Depression Scale for schizophrenia^69^ was used to assess depression and is known to be reliable in CHR individuals.^70^ A total score between 0 and 36, was used to reflect the sum of 9 depression items scored across the previous 2 weeks. Daily stress was measured using the daily stress inventory.^71^ A total score between 0 and 406, was used to reflect the sum of 58 items scored on a 7-point Likert scale across the previous 24 hours. SIPs severity was measured using the continuous SIPs positive scale, which is calculated as the sum of the 5 positive severity items, with scores ranging from 0 to 6, meaning total scores range between 0 and 30.

### Statistical Analyses

Samples were split into 2 groups, those meeting COGDIS criteria (COGDIS+) and those not meeting criteria (COGDIS−). To explore differences between these groups, a Pearson’s chi-squared test was used to explore variation across sex and transition rate. A Fischer’s exact test was used to explore variation across ethnicity and at-risk-mental-state groups. The Kolmogorov–Smirnov test was used to test normality for age, depression, and stress, none of which were normally distributed. A Wilcoxon’s signed rank sum test was used to explore variation across age, depression, and stress.

Before running further statistical tests, we were interested in comparing CHR samples across 3 distinct diagnostic groups, those with a DSM-5 diagnosis of either SPD, ASD, or BPD. One participant screened positive for both SPD and ASD, and 7 participants screened positive for both SPD and BPD. To avoid any statistical bias, these 8 individuals were removed from subsequent statistical analyses. Multivariate logistic regression analyses were then performed for each group, using COGDIS status, age, and SIPs severity as predictors, to examine whether basic symptoms were predictive of diagnosis.

As a sensitivity analysis, we investigated the relationship between COGDIS and SIPs positive/negative/disorganized severity across the full sample, as well as within each of the diagnostic sub-samples (SPD/ASD/BPD). We calculated a dimensional COGDIS score from the total number of endorsed items, then calculated the Spearman correlation with SIPs severity scores (see Supplementary Material). Analyses were performed using R 4.3.1.^72^ All tables were developed using the “gtsummary” package.^73^

## Results

Of the 685 CHR participants who completed the COGDIS items of the SPI, a total of 452 met additional COGDIS criteria (COGDIS+), whereas 233 CHR participants did not meet COGDIS criteria (COGDIS−). A total of 68 participants transitioned to psychosis within 24 months, an overall transition rate of 10%. Baseline demographics are listed in Table 2.

**Table 2.** Baseline Demographics and Clinical Characteristics of Individuals at Clinical High Risk for Psychosis (CHR) With (COGDIS+) or Without (COGDIS−) Meeting Additional COGDIS Criteria (*N* = 685)

Variable	Does Not Meet COGDIS Criteria, (*N* = 233)^1^	Meets COGDIS Criteria (*N* = 452)^1^	*P*-value^2^
Sex at birth (female)	50%(116/233)	43%(196/452)	.11
Age in years	18.10 ± 3.86	19.02+/-4.13	.003
Ethnic group			.3
Unknown	0%(0/233)	0.2%(1/452)	
American Indian/Alaska native	1.3%(3/233)	2.4%(11/452)	
Asian	12%(29/233)	11%(50/452)	
Black or African American	11%(25/233)	12%(54/452)	
More than one race	15%(36/233)	12%(55/452)	
Native Hawaiian or Pacific Islander	0.9%(2/233)	0%(0/452)	
White	59%(138/233)	62%(281/452)	
Years of education completed	10.97 ± 2.95	11.71 ± 3.09	.003
Estimated current IQ	104.77 ± 15.43	105.96 ± 16.25	.2
Meets criteria for an at-risk-mental-state group			.11
Genetic risk and deterioration (GRD)	0.9%(2/232)	0.7%(3/452)	
Attenuated positive symptoms (APS)	97%(224/232)	94%(425/452)	
APS and GRD	1.7%(4/232)	4.9%(22/452)	
Brief intermittent positive symptoms (BIPS)	0.9%(2/232)	0.2%(1/452)	
APS and BIPS	0%(0/232)	0.2%(1/452)	
Active medications			
None	65% (151/232)	54% (244/452)	
Antidepressant	36% (84/232)	44% (199/452)	
Antipsychotic	34% (78/232)	40% (182/452)	
Benzodiazepines	34% (79/232)	40% (181/452)	
Total depression score	4.59 ± 4.08	7.21 ± 4.36	<.001
Transition to psychosis	6%(14/233)	11.9%(54/452)	.01
Daily stress score	63.73 ± 52.16	72.24 ± 51.83	.010

^1^%(*n*/*N*); mean±SD(*N*).

^2^Pearson’s Chi-squared test; Wilcoxon rank sum test; Fisher’s exact test.

Within the COGDIS− group, 50% of participants were female, 59% of participants were of ‘white’ ethnicity, and mean age was 18.10 years (SD = 3.86). Within the COGDIS+ group, 43% of participants were female, 62% of participants were of ‘White’ ethnicity, and there was a mean age of 19.02 years (SD = 4.13), (*P* = 0.003). Six percent of participants in the COGDIS− group and 11.9% of participants in the COGDIS+ group transitioned to psychosis, (*P* = .01). The mean depression score was 4.59 (SD = 4.08) in the COGDIS− group and 7.21 (SD = 4.36) in the COGDIS+ group, (*P* < .001). The mean stress score was 63.73 (SD = 52.16) in the COGDIS− group and 72.24 (SD = 51.83) in the COGDIS+ group, (*P* = .01).

### Characteristics of Participants With SPD, ASD, or BPD in NAPLs-3

For our primary analysis, we were interested in comparing COGDIS scores between CHR and CHR with additional comorbid diagnoses of either SPD, ASD, or BPD. After removing the 10 participants who met diagnostic criteria for more than one of SPD, ASD, and BPD, the remaining sample of 675 participants consisted of 68 individuals who screened positive for SPD, 18 individuals who screened positive for ASD, 21 individuals who screened positive for BPD, and 568 participants who did not meet any of the 3 comorbid diagnostic criteria. Clinical characteristics are listed in Table 3. Those meeting criteria for BPD were predominantly female, were older and had more years of education in comparison to those with SPD and ASD. Those meeting criteria for ASD were predominantly male, and the youngest, with the least years of education. Mean depression score was highest 7.43 (SD = 4.80) in the BPD group, followed by 7.26 (SD = 4.82) in the SPD group, and 4.11 (SD = 3.72) in the ASD group (*P* = .029). Transition rate was highest in SPD at 22% followed by, 11% in the ASD group, and 5% in the BPD group however these differences did not reach significance.

**Table 3.** Baseline Demographics and Clinical Characteristics of Individuals at Clinical High Risk for Psychosis (CHR) With Additional Diagnosis of Either SPD, ASD, or BPD (*N* = 675)

Variable	Meets DSM-5 Criteria for Schizotypal Personality Disorder, *N* = 68^1^	Meets DSM-5 Criteria for Autism Spectrum Disorder, *N* = 18^1^	Meets DSM-5 Criteria for Borderline Personality Disorder, *N* = 21^1^	*P*-value^2^
Sex at birth (female)	37%(25/68)	44%(8/18)	76%(16/21)	.006
Age in years	20.30 ± 4.41	15.93 ± 2.49	22.13 ± 4.24	<.001
Years of education completed	12.36 ± 2.60	9.44 ± 2.53	14.00 ± 3.27	<.001
Estimated current IQ	103.52 ± 17.67	112.59 ± 16.24	106.95 ± 15.70	.2
Meets criteria for an at-risk-mental-state group				.2
Genetic risk and deterioration (GRD)	2.9%(2/68)	0%(0/18)	0%(0/21)	
Attenuated positive symptoms (APS)	85%(58/68)	100%(18/18)	100%(21/21)	
APS and GRD	12%(8/68)	0%(0/18)	0%(0/21)	
Brief intermittent positive symptoms (BIPS)	0%(0/68)	0%(0/18)	0%(0/21)	
APS and BIPS	0%(0/68)	0%(0/18)	0%(0/21)	
Total depression score	7.26 ± 4.82	4.11 ± 3.72	7.43 ± 4.80	.029
Transition to psychosis	22%(15/68)	11%(2/18)	5%(1/21)	.14
Daily stress score	75.62 ± 49.20	69.35 ± 57.95	77.90 ± 55.56	.7

^1^%(*n*/*N*); mean ± SD(*N*).

^2^Fisher’s exact test; Kruskal–Wallis rank sum test.

### Basic Symptoms in NAPLs-3 Participants With a Diagnosis of SPD, ASD, or BPD

A logistic regression model was then used to analyze the relationship between COGDIS criteria and diagnostic criteria for SPD, ASD, or BPD while controlling for age. Meeting COGDIS criteria was positively associated with SPD diagnosis (OR = 1.72, CI = [1.31–2.28], *P* = .001). In contrast, COGDIS criteria were not associated with a diagnosis of ASD or BPD in a CHR sample (Table 4). Similarly, those with more severe positive symptoms were more likely to meet the criteria for SPD but did not predict criteria for BPD or ASD. Additionally, age varied significantly across diagnoses, such that older participants were more likely to meet the criteria for SPD and for BPD, and younger participants were more likely to meet criteria for ASD.

**Table 4.** Likelihood of Meeting DSM-5 Diagnostic Criteria Based on COGDIS Criteria (*N* = 685)

	Meets DSM-5 Criteria for Schizotypal Personality Disorder (*N* = 68)			Meets DSM-5 Criteria for Autism Spectrum Disorder (*N* = 18)			Meets DSM-5 Criteria for Borderline Personality Disorder (*N* = 21)		
Characteristic	OR^1^	95% CI^1^	*P*-value	OR^1^	95% CI^1^	*P*-value	OR^1^	95% CI^1^	*P*-value
Meets COGDIS criteria	1.72	1.31, 2.28	<.001	0.73	0.48, 1.10	.13	0.86	0.59, 1.26	.4
Age	1.11	1.08, 1.14	<.001	0.86	0.81, 0.92	<.001	1.17	1.13, 1.22	<.001
SIPS positive	1.10	1.07, 1.13	<.001	1.03	0.99, 1.08	.14	1.02	0.99, 1.06	.2

^1^OR = odds ratio, CI = confidence interval.

In the full sample, a moderate positive correlation was found between COGDIS and SIPs positive, negative, and disorganized severity scores. This relationship was no longer present in the SPD or ASD subsample. In the BPD group, a moderate positive correlation was found between COGDIS and SIPs positive severity (see Supplementary Material).

## Discussion

Our primary aim was to investigate the value of COGDIS in delineating among SPD, ASD, and BPD in CHR services. Endorsing COGDIS criteria was associated with an increased likelihood (OR = 1.72, *P* < .001) of meeting diagnostic criteria for SPD. In contrast, COGDIS was not associated with ASD or BPD. Our findings suggest that in this CHR sample, COGDIS identifies subtle subjective experiences that vary across SPD, ASD, and BPD and are independent of the severity of prodromal psychotic symptoms. We also found that participants meeting COGDIS criteria on the whole had a higher rate of transition to psychosis as well as significantly greater daily stress and depressive symptoms. Moderate positive correlations were found between COGDIS and SIPs severity scores across positive, negative, and disorganized domains. Of note, these relationships were no longer significant in the diagnostic sub-samples, with the exception of positive symptom severity in the BPD group. This is particularly interesting in the SPD sub-sample, however small sample size prevents further interpretation, particularly in the ASD and BPD groups.

In this sample, 10% of participants met the criteria for SPD, whereas 4% met the criteria for BPD, and 3% for ASD. Several meta-analyses report that prevalence of SPD, BPD, and ASD in CHR-P are approximately 12%, 10%, and 4%, respectively^10,12,74^ and that prevalence among CHR-P samples are notably heterogenous.^10^ In the context of the documented rise in ASD worldwide,^75–78^ comorbidity between ASD and SPD may also become more common. In this sample of youth identified as CHR, 25% of the BPD group and 5% of the ASD group also met SPD criteria.

A growing literature places “basic self-disturbance” centrally in the pathophysiology underlying SSD’s^79^ and our findings support the notion that one fundamental difference between SSDs and other forms of psychopathology, is a disturbance of “basic self.”^47^ Authors have also drawn attention to the concern that SPD is a neglected diagnosis.^24^ For example, in a study of 30 patients diagnosed with BPD, approximately 46% met criteria for SPD and 20% for schizophrenia. These individuals scored higher on a measure of “basic self-disturbance.”^80^

Similarly, in DSM-5’s diagnostic hierarchy, a diagnosis of SPD is excluded where a previous diagnosis of ASD has already been made, yet some people with a valid diagnosis of autism in childhood may go on to develop comorbid features of SPD without developing full blown psychosis.^81^ Here, subjective COPER discrepancies more associated with SPD are overlooked as they are missing from the mechanisms of categorization,^24^ leaving many young people with inadequate explanatory models of their experience. The CER study, among others,^33,63^ has demonstrated the value of COGDIS in predicting the onset of schizophrenia, as defined by first-rank psychotic features. Our findings suggest that COGDIS may have additional value in detecting the underlying diagnostic features specific to schizophrenia in a sample where all participants display prodromal features.

The benefits of early intervention are increasingly recognized in CHR, in parallel with the development of early psychosis services. Young people often attend CHR services with a complex presentation of attenuated psychotic symptoms and pre-existing ASD or BPD diagnoses, alongside a pattern of social risk factors, and no history of disease trajectory. Determining which treatment strategies will be effective is therefore notoriously challenging with limited knowledge of the underlying psychopathology. Within CHR services specifically, broad symptom domains of psychosis, such as hallucinations, delusions, and thought disorganization, may be less sensitive to subtle subjective experiences,^82–84^ particularly aspects of self-experience.^9^ In practice, this means that clinical decision-making has predominantly relied on observable behavioral traits.^85^

Now, for the first time in a CHR sample, we demonstrate the potential discriminative value of basic symptoms and suggest that COGDIS be utilized in CHR services to inform clinicians who wish to determine which patients may benefit from supplementing “CHR treatment” with additional schizophrenia specific strategies. For example, there is some evidence for low dose antipsychotic use in managing positive symptoms in SPD.^86^ Similarly, psychoeducation and cognitive remediation may be important in the management of cognitive symptoms in SPD as opposed to ASD or BPD^87^ and a randomized controlled trial of a combined pharmacological and psychosocial intervention found promising results in alleviating general symptoms in SPD.^88^

A higher rate of transition to psychosis was also found in those meeting COGDIS criteria. The prevalence rate of COGDIS was higher in our study (66%) than the wider UHR/CHR literature (50%),^44,89^ yet the clinical characteristics of those meeting COGDIS criteria are in line with previous findings.^90^ A recent UHR study in Australia, found a significant association only between COGDIS total score and transition and this relationship was lost when controlling for measures of UHR severity.^44^ Hence, the utility of COGDIS as an additional predictive tool within a UHR/CHR sample is questionable. In contrast, our findings^55^ suggest that COGDIS can help detect those within the CHR sample who experience “basic self-disturbance,” more likely associated with an underlying SSD, as opposed to neurodivergent or borderline personality comorbidities. Further investigation into the precise role of COGDIS within CHR may be warranted.

We also acknowledge some limitations of our study. First, these results should be interpreted cautiously because of small sample sizes in the ASD and BPD groups. We also note that groups varied in sex and years of education, but these variables were not included in regression modeling due to small sample size. However, in a US-based sample with mean age of 18-years, variation in education likely reflects the average age difference across the COGDIS−/COGDIS+ groups. Sex differences cannot be accounted for in this way, though there is no evidence of sex differences in COGDIS and prevalence rates across comorbidities are consistent with epidemiological studies of ASD, SPD, and BPD.^29^

Second, we chose to use SCID-5 diagnoses as our outcome measure because these are well-recognized criteria designed to delineate disorders above a clinically meaningful threshold. Relying on dichotomous classifications limits any dimensional investigation of psychopathological spectra and we recommend that future studies include both diagnostic and dimensional indicators.

Improved diagnostic processes would also further enhance our findings. Several studies report excellent clinical validity and reliability across SCID-5 modules,^91–93^ yet SCID-5 may be less sensitive to personality disorders, such as SPD and BPD.^94^ Although a specific SCID-5 personality module exists, the time required to investigate each of the 10 recognized personality disorders via clinical interview makes its use often impractical in both research and clinic. Similarly, a robust diagnosis of ASD requires assessment tools such as the autism diagnostic observation schedule,^21^ alongside family history and medical records. Such procedures are rarely feasible in large datasets.

Third, in this sample, young persons with recognized intellectual impairment were excluded. BS may be more challenging to detect in those with lower verbal ability, as they are less able to verbalize the “odd” subjective disturbances that characterize BS.^95,96^ For a similar reason, BS are not reliably detectable in those below 8 years of age. Notably, young persons with intellectual impairment disproportionately present at CHR services and constitute 25% of those with ASD.^97,98^ Due to the design of this study, our findings cannot be generalized to this group.

This research was a secondary analysis of the NAPLS-3 dataset. Alongside COGDIS, COPER basic symptoms capture complementary aspects of “basic self-disturbance” and psychosis risk. However, only COGDIS items from the SPI-A/CY were collected as part of the NAPLS-3 protocol. While we acknowledge this as a limitation, we also stress that transition risk was not part of our primary analysis.

Similarly, while COGDIS captures aspects of “basic self-disturbance,” the phenomenon is probably best captured by the EASE. When comparing the detection of SD across both BS and EASE, Burgin et al.^50^ report effect sizes of *g* = 0.77 and *r* = 1.60, respectively. Furthermore, a systematic review of “basic self-disturbance” omitted research utilizing BS in recognition of the greater validity of the EASE,^48^ while acknowledging that the SD construct emerged from BS.

Notwithstanding these differences, a recent factor analysis of the EASE concluded that SD may be best characterized by a dominant single factor of disturbed subjectivity with substantial variability across items.^49^ While COGDIS may not be fully representative of SD, the author noted that many EASE items are nonspecific to the schizophrenia spectrum.

In routine clinical practice, extensive phenomenological interviews such as EASE are more challenging to widely adopt because of the time required for administration. Concurrently, sample size is often a substantial limitation of research in this area.^79^ In contrast, a 9-item interview such as COGDIS can be administered within 10–15 minutes and is able to detect basic symptoms, in those as young as 8 years-old.^66^

This study aimed to investigate the discriminative value of COGDIS in differentiating among SPD, ASD, and BPD, in a CHR sample. Meeting COGDIS criteria was associated with an increased likelihood of reaching diagnostic criteria for SPD, whereas COGDIS was not associated with ASD or BPD. Our findings corroborate previous studies which propose a fundamental difference across spectra at the level of the “basic self,” and that these disturbances can be captured by COGDIS. The utilization of COGDIS in clinical practice may address some of the challenges facing the CHR/UHR approach and become a useful tool for informing disorder-specific treatment strategies in CHR-P services.

## Supplementary Material

sgae017_suppl_Supplementary_Table
