# Supplementary material for: A Tale of Three Spectra: Basic Symptoms in Clinical-High-Risk of Psychosis Vary Across Autism Spectrum Disorder, Schizotypal Personality Disorder, and Borderline Personality Disorder
Source: Schizophr Bull Open. 2024 Jul 27;5(1):sgae017. doi: 10.1093/schizbullopen/sgae017 (PMC11341945; doi:10.1093/schizbullopen/sgae017)
Supplement: sgae017_suppl_Supplementary_Table [file sgae017_suppl_supplementary_table.docx]

***Table S.1. Spearman correlations showing relationships between COGDIS and Structured Interview for Psychosis-risk Syndromes (SIPs) domains across the NAPLS-3 sample***

|  | **SIPs Positive Severity**^+^ | **SIPs negative severity**^+^ | **SIPs disorganized severity**^+^ |
| --- | --- | --- | --- |
| ^1^**COGDIS** |  |  |  |
| ^2^CHR (*N=685)* | 0.35*** | 0.39*** | 0.39*** |
| ^3^CHR + SPD (*N=68)* | 0.16 | 0.18 | 0.20 |
| ^3^CHR + ASD (*N=18)* | 0.39 | 0.40 | 0.21 |
| ^3^CHR + BPD (*N=21)* | 0.42* | 0.36 | 0.28 |
| *p<0.05, **p<0.01, ***p<0.001, ^+^spearman correlation, ^1^total number of endorsed COGDIS items, ^2^Clinical High Risk, ^3^participants meeting CHR criteria and comorbid diagnosis of either SPD, ASD, or BPD | | | |
